# Machine Learning Method and Hyperspectral Imaging for Precise Determination of Glucose and Silicon Levels

**DOI:** 10.3390/s24041306

**Published:** 2024-02-18

**Authors:** Adam Wawerski, Barbara Siemiątkowska, Michał Józwik, Bartłomiej Fajdek, Małgorzata Partyka

**Affiliations:** Faculty of Mechatronics, Warsaw University of Technology, Sw. A. Boboli St. 8, 02-525 Warsaw, Poland; adamwawerski30@gmail.com (A.W.); michal.jozwik@pw.edu.pl (M.J.); bartlomiej.fajdek@pw.edu.pl (B.F.); malgosiapartyka@gmail.com (M.P.)

**Keywords:** hyperspectral imaging, artificial intelligence, health monitoring, glucose monitoring, silicon monitoring

## Abstract

This article introduces an algorithm for detecting glucose and silicon levels in solution. The research focuses on addressing the critical need for accurate and efficient glucose monitoring, particularly in the context of diabetic management. Understanding and monitoring silicon levels in the body is crucial due to its significant role in various physiological processes. Silicon, while often overshadowed by other minerals, plays a vital role in bone health, collagen formation, and connective tissue integrity. Moreover, recent research suggests its potential involvement in neurological health and the prevention of certain degenerative diseases. Investigating silicon levels becomes essential for a comprehensive understanding of its impact on overall health and well-being and paves the way for targeted interventions and personalized healthcare strategies. The approach presented in this paper is based on the integration of hyperspectral data and artificial intelligence techniques. The algorithm investigates the effectiveness of two distinct models utilizing SVMR and a perceptron independently. SVMR is employed to establish a robust regression model that maps input features to continuous glucose and silicon values. The study outlines the methodology, including feature selection, model training, and evaluation metrics. Experimental results demonstrate the algorithm’s effectiveness at accurately predicting glucose and silicon concentrations and showcases its potential for real-world application in continuous glucose and silicon monitoring systems.

## 1. Introduction

As medicine advances, modern technologies are increasingly emerging to diagnose various parameters of our body non-invasively. One such innovative approach is the use of a hyperspectral camera.

Hyperspectral imaging involves capturing and processing information from a wide range of electromagnetic wavelengths, providing detailed spectral information for each pixel in an image. The complexity of hyperspectral data makes it challenging to analyze. One of the primary roles of AI in hyperspectral data analysis is spectral unmixing. This process involves separating the contributions of different materials or substances present in a scene based on their spectral signatures. Classification is another critical application of AI in hyperspectral image analysis. By training machine learning models on labeled hyperspectral datasets, AI algorithms can learn to distinguish between different classes of materials. This capability is invaluable in various fields, including agriculture, environmental monitoring, and remote sensing, where precise identification of objects or substances is essential.

This article focuses on the use of a hyperspectral camera to measure glucose and silicon levels. Focusing on the measurement of glucose levels is particularly relevant because of its key role in metabolic processes and the associated implications for people with diabetes. At the same time, the study of silicon levels opens up a new area of exploration to understand the potential impact of this element on human health. Silicone (Si) is the third most abundant element (after iron and zinc) in the human body. It is mainly concentrated in connective tissue—aorta, trachea, tendons, bones, and skin. In bones, silicone binds with glycosaminoglycans and forms (together with collagen and proteoglycans) an extracellular matrix. Studies show that silicone plays an important role during the process of active growth and bone mineralization. The specific mechanism is not yet well understood but is likely related to collagen synthesis and stabilization in bone matrix. Silicone’s importance in bone regeneration and maintaining proper bone mineral density has also been underlined by scientists [1].

### 1.1. Silicone (Si)

Some studies suggest that silicon may support bone health and counteract bone loss associated with osteoporosis. It plays a role in collagen production, which is essential for joint health. A higher level of silicon may contribute to maintaining joint flexibility and proper joint function. Research suggests that a deficiency in silicon may affect immune functions.

Silica exists in different forms; however, only one-orthosilicic acid [Si(OH)_4_] is absorbed by the human organism [2]. Fluids such as drinking water and beer have been considered the best sources of silicone in its bioavailable form. Solid food contains silica that can be hydrolyzed during the process of digestion. Rice, breakfast cereals, breads, and pasta are particularly good sources of silicone [3]. Consequently, in countries such as China or India, where a plant-based diet is prevalent, silicone intake is much higher than in the West. Interestingly, it has been shown that the incidence of hip fracture is lower in countries where silicone intake is higher [1]. Besides its role in connective tissue formation, stabilization, and bone formation [4], silicone’s role in the processes of immune or inflammatory response and influence on cognitive functions and copper and magnesium absorption have been described [4]. Daily minimum requirements for silicon might vary and depend on age and sex. There is no recommended daily intake for this trace element. However, studies show that 10–25 mg silicone should be provided each day to balance its urinary excretion [5].

Since silicone intake is linked with optimal bone formation and maintaining bone density, non-invasive monitoring of its levels is particularly important in small children and elderly people.

### 1.2. Related Works—Silicone (Si)

While we can find many methods in the literature where non-invasive methods for detecting glucose levels are described, methods for describing silicon levels are not reported. In our study, we attempt to fill this gap. Moreover, we prove with our proposed analysis methods that it is possible to extract precise information about the concentration of the substance of interest.

### 1.3. Glucose (Glc)

We observe a continuous upward trend in the incidence of diabetes. We can say that we are currently dealing with an epidemic of this disease. In 1985, the WHO estimated that 200 million people would have diabetes in the next 15 years. It is indisputable that glucose control is crucial for maintaining overall health, especially for individuals with diabetes and those at risk of developing diabetes [6,7,8]. People with both type 1 and type 2 diabetes have a higher likelihood of developing cardiovascular disease (CVD) and facing an increased risk of mortality. This elevated risk results in a reduction of their life expectancy by approximately 5 to 15 years [9]. For this reason, it is indisputable that it is necessary to constantly improve methods for monitoring blood glucose levels in people at increased risk.

### 1.4. Related Works—Glucose (Glc)

Numerous techniques have been explored for non-invasive glucose sensing using hyperspectral imaging. A comprehensive overview of the methods is presented in [10]. Table 1 summarizes the articles relevant to the available methods for non-invasive glucose detection. For each item, the main goals, advantages, disadvantages and method used are presented.

Infrared (NIR) spectroscopy operating in the 750–2500 nm wavelength range is a cost-effective and compact method for quantifying glucose levels in biological tissue [12,17]. Mid-infrared (MIR) spectroscopy gathers numerical information about a sample based on its absorption spectra. However, MIR employs a longer wavelength of light, specifically in the 2500–10,000 nm range, covering the fingerprint region of glucose. This results in reduced scattering and higher absorption in the tissue, leading to distinct and sharp peaks in the absorption spectra of glucose and other chromophores in contrast to the weak and broad peaks observed in the NIR band response [18].

The Raman technique employs monochromatic light spanning from visible to MIR to assess glucose concentration through the Raman effect [15].

Far-infrared (FIR) spectroscopy is a technique that deals with the study of the absorption, emission, and reflection of infrared radiation in the far-infrared region of the electromagnetic spectrum. Strong water absorption and limited penetration make glucose estimation challenging [19].

In the paper [20], the authors specifically explored the feasibility of detecting glucose concentrations with optical coherence tomography (OCT). The research demonstrated OCT’s potential for detecting glucose concentrations in whole blood. The method exhibited a notable differentiating resolution for various glucose concentrations, with the ability to detect differences as small as 65 mg/dL. However, the resolution was significantly impacted when dealing with samples with a significant multiple scattering component.

Dynamically developing hyperspectral cameras can also deliver a large amount of data of the functions of wavelengths from the examined sample. Additionally, quantitative statistical methods or machine learning algorithms such as support vector machine (SVM) or artificial neural networks (ANNs) can be employed to obtain the required sample parameters [21]. Hyperspectral imaging and artificial intelligence (AI) integration have emerged as a promising avenue for non-invasive analysis and monitoring of various substances. Studies presented in [22,23] have demonstrated the effectiveness of AI for extracting relevant features from hyperspectral data and improving accuracy in material identification and concentration estimation.

The paper [24] presented the possibility of dissecting the concentration of glucose in deionized water. This paper investigates six machine learning methods for accurately predicting and identifying significant absorbance wavelengths for glucose aqueous concentrations measured through NIR spectroscopy. Notably, SVM, deep learning, and PCA-NN (the bucketed PCA neural network) models demonstrate good prediction quality. The study emphasizes the importance of feature pattern analysis for understanding model performance and for revealing essential features within specific wavelength regions.

### 1.5. Introduction Remarks

Our work proposes the use of a hyperspectral camera to determine the levels of chemical compounds. The work is preliminary: we are investigating the concentrations of glucose and silicon in distilled water. By using modern artificial intelligence methods, we have been able to achieve promising results.

The paper presented here consists of the following parts: After an introduction, we present a literature review related to the detection of glucose and silicon levels. In Section 2, we present a description of the hyperspectral camera and the methodology of our approach. In the next section, we describe the experimental results. The article concludes with a summary and bibliography. The data collected for the study are available at [25].

## 2. Materials and Methods

### 2.1. Hyperspectral Imaging

Hyperspectral imaging (HSI) is a method of capturing and processing images that are represented by electromagnetic radiation in the near-infrared (NIR), visible light (VIS), and ultraviolet (UV) ranges [26]. This technique is applied in various industries where particle recognition, material identification, and analysis of substance compositions and concentrations are required.

The initial step in HSI involves illuminating the target element with a light source that covers the spectral range of the hyperspectral camera. Halogen lamps are often used for this purpose due to their low cost compared to their high spectral power density. After the reflection from the sample, the light beam is collected by an objective and passes through the entrance slit. The slit is designed to only allow light from a narrow linear region to pass through. After collimation, the light is dispersed into a sequence of narrow spectral bands and is visualized on a CMOS matrix detector. The collected image represents a single line of the object as a function of its wavelength [27].

To obtain a complete image of the sample, it is essential to correlate the acquisition of the line with the sample’s linear translation. Dedicated linear tables provided by the camera manufacturer and with adjustable table movement lengths and speeds are often used for this purpose. By scanning individual lines, the hyperspectral camera eventually accumulates an image that is the sum of all the lines. The resulting image is often presented using a hyperspectral cube encompassing both spatial and spectral dimensions, which facilitates a comprehensive representation of the analyzed sample’s properties.

Hyperspectral devices for imaging capture the intensity, which is denoted as: I(x,y,λ), where *x* and *y* are pixel coordinates, λ represents the wavelength of electromagnetic radiation, and *I* is the radiation intensity. Additionally, the quantities Δx,Δy determine the spatial resolution of the image and are directly dependent on the camera’s construction, including the lenses and detectors used. The size Δλ corresponds to the spectral resolution, i.e., the intervals between consecutive spectra—this size is known as the full width at half maximum (FWHM) [28].

Hyperspectral imaging provides continuous intensity information for the entire camera spectrum, unlike multispectral imaging, for which intensity is discretely characterized. The collected hyperspectral data are represented by a matrix $X\in R^{M\times N\times L}$, where dimensions M represent the length, N is the width of the image, and L is the number of all channels of light wavelengths.

Hyperspectral cameras find extensive application in various industries and in material science. They are utilized for:

- Conducting research on plant phenotyping [29];
- Analyzing artwork and historical artifacts for identifying material compositions used in paintings [30];
- Material science, particularly in waste sorting facilities for waste classification [31];
- The medical field for examining tissues and pathological changes [32].

In hyperspectral imaging, the sample’s content can be studied based on the scattering, absorption, or rotation of the plane of the electromagnetic waves. The detection of particle concentrations through the scattering of rays in the near-infrared range is associated with challenges related to the precision and repeatability of measurement results. Factors like varied human body temperatures causing changes to the scattering of incoming radiation to the camera [33] and intensity changes resulting from scattering are within the range of measurement noise [34] and are a source of the noise.

Due to the above factors, a more favorable approach is to apply a detection method based on the examination of radiation absorption.

The research project focuses on non-invasive glucose and silicon level detection using hyperspectral imaging techniques. The absorption of radiation originating from glucose falls within similar wavelength intervals as water, hemoglobin, and fats [35]. Through application of appropriate data analysis methods, it is possible to extract information about the concentration of the molecule of interest.

The research project focuses on non-invasive glucose level detection using hyperspectral imaging techniques. The study progresses through a systematic series of steps and data inspections aimed at achieving accurate and reliable results.

### 2.2. Data Acquisition

To obtain reliable data, a series of methodological steps were taken to ensure the best conditions and repeatability for the collected samples. To increase the diversity of data, the data acquisition process was carried out in two separate sessions with a one-month interval between.

#### Sample Preparation

The initial stage involved preparing controlled samples. A diverse set of solutions was prepared for the study and included different concentrations of glucose in saline solution and distilled water.

Additionally, silicon (Si) in distilled water was chosen as a material in order to study our ability to detect its concentration. The aim was to create an initial representative range of materials that mimic complex and diverse scenarios encountered in real-world applications.

### 2.3. Measurement Setup

**Figure 1:**
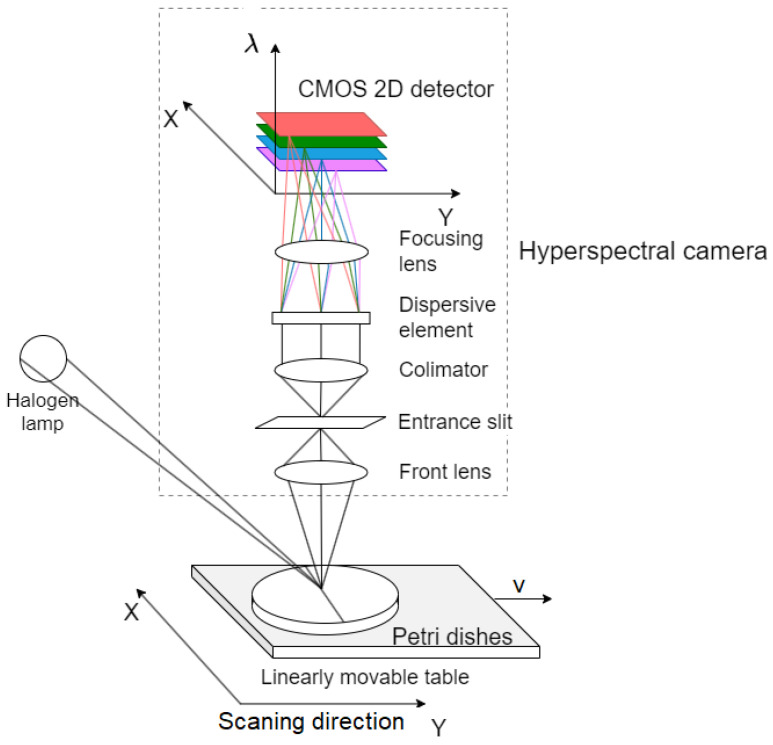
Data collection system.

The measurement setup was equipped with a hyperspectral camera (Headwall, model: Micro-Hyperspec SWIR 384, wavelength range: 900–2500 nm) mounted vertically over a linearly movable table. The samples were placed on the table and illuminated with a halogen lamp. Each of the prepared solutions was poured into Petri dishes made of polystyrene (PS). Figure 1 presents a testbed for data collection.

The next step was to set the focus of the lens depending on the height of the placed camera. Headwall provides the software necessary for data acquisition for their devices. In the software, parameters such as the speed and distance of linear table movement and the camera’s operating parameters, such as exposure intervals for each line of the captured image and the duration of data acquisition by the camera, needed to be set. The data collected for the study are available at [25].

#### 2.3.1. Initial Data Inspection for Glucose in Distilled Water

The whole dataset contains 94,730 samples of all glucose concentrations, and for silicon, there are a total of 47,375 samples. The obtained data were initially processed to classify their quality and explore the potential for predicting data based on them. The whole dataset’s hyperspectral data for all glucose concentrations is equal.

From the determined parameters for each wavelength, it can be inferred that the dataset has:

- Spectral data range from 892.96nm to 2506.03nm in intervals of approximately 9 nm between each channel.
- Spectral data are stored in the int64 format.
- There are 94,730 samples for each wavelength.
- The skewness coefficient for the entire wavelength range is positive and ranges from 0.2 to 8.5, excluding the wavelength 902.503 nm.
- Kurtosis for wavelengths in the range of 902.503–1312.93 nm and 2315.13–2506.03 nm has values from −0.2 to −1.7, while for wavelengths 1389.29–1580.18 nm, it ranges from 70 to 163. For wavelengths 1608.82–2019.24 nm, this coefficient is between 25 and 55, and for the remaining wavelengths, it ranges from 0.2 to 5.
- The linear correlation coefficient between the wavelength intensity value and glucose concentration using the Pearson method is approximately −0.3.

The correlation coefficient indicates the convergence of two variables, i.e., the possibility of predicting one variable based on the other. In this case, it is useful information for predicting the concentration of particles using wavelengths.

The absolute value of the skewness coefficient ranges from 0.1 to 9. A high absolute value of the skewness coefficient indicates higher asymmetry of the studied samples and a lack of good repeatability of the collected data by the camera for these wavelengths. Positive values indicate rightward skewness, while negative values indicate a leftward shift relative to the system. For a normal distribution, it is 0.

For a normal distribution, the kurtosis coefficient is 3, which indicates a normal distribution of measurement data. A coefficient below this value is associated with a platykurtic distribution, which is flatter than a normal distribution. Characteristics of such a distribution include equal placement of points around the mean and a lower probability of outlier values compared to other cases. When the value is exceeded, the data are more concentrated around the mean, and the distribution has a leptokurtic character [36]. High kurtosis in the area of light absorption may indicate a higher occurrence of extreme values associated with the absorption of energy for these wavelengths and thus indicate the possibility of changing the intensity of light intensity at different glucose concentrations.

Detailed statistical information is presented in Table 2 (glucose) and Table 3 (silicon). The values of individual parameters such as standard deviation, skewness, kurtosis, and correlation for different wavelengths are presented to provide insights into different aspects of a dataset.

Additionally, the correlation matrix between the concentration and each wavelength was determined using the formula:

(1) $$r=\sum_{i=1}^{p}\frac{\left(x_{i}-\mu_{x}\right)\cdot \left(y_{i}-\mu_{y}\right)}{\left(p-1\right)\sigma_{x}\sigma_{y}},$$

where: *p*—the number of pixels in vector X, $\mu_{x}$—the mean for a single spectral channel, and $\sigma_{x}$—the standard deviation for a single spectral channel.

The wavelengths were also compared to investigate their correlation and linear dependencies. It is visible in the plot that most of the data may introduce disturbances to the model. Dimensionality reduction of hyperspectral data would be beneficial.

**Figure 2:**
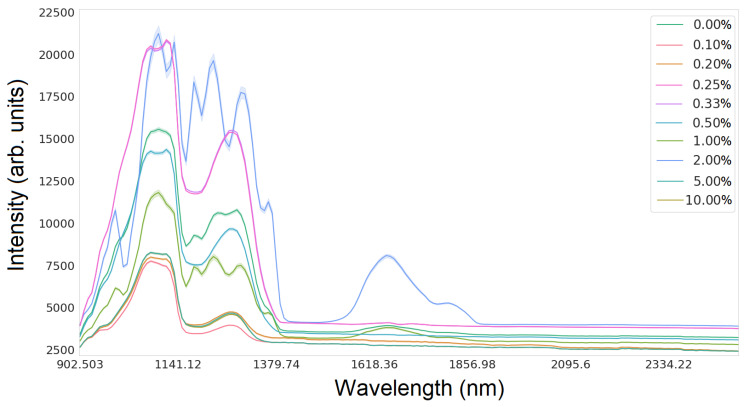
Lineplot of hyperspectral data for all glucose concentrations in the wavelength range from 892 nm to 2506 nm.

**Figure 3:**
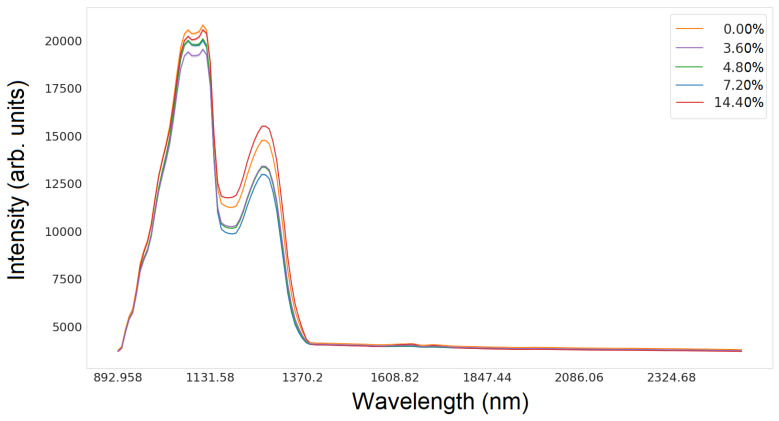
Lineplot of hyperspectral data for all silicon concentrations in the wavelength range from 892 nm to 2506 nm.

Figure 2 and Figure 3 show the intensity values depending on the wavelength for glucose and silicon, respectively.

The examined parameters represent statistical features that impact the initial data preprocessing, detection of outliers, feature selection, and modeling decisions. Thorough consideration of these statistics can lead to more accurate and significant analyses and conclusions from the hyperspectral dataset. For the collected data, the kurtosis coefficient ranges from 0 to 100, indicating a diverse distribution of data for each channel. For wavelengths for which the intensity values are constant, the kurtosis coefficient falls within the <−2.0> range. The highest values were observed for the wavelength range from 1150 nm to 1700 nm. The correlation coefficient between the intensity of light and the glucose concentration is approximately −0.3, indicating a decreasing linear relationship between these variables.

Due to erroneous values for the wavelength 892.958 nm, they seem unreliable. Errors for a given wavelength may result from damage to the device or overexposure at that wavelength. It would be necessary to remove data for this wavelength to ensure the overall quality of the dataset.

#### 2.3.2. Preprocessing

**Figure 4:**
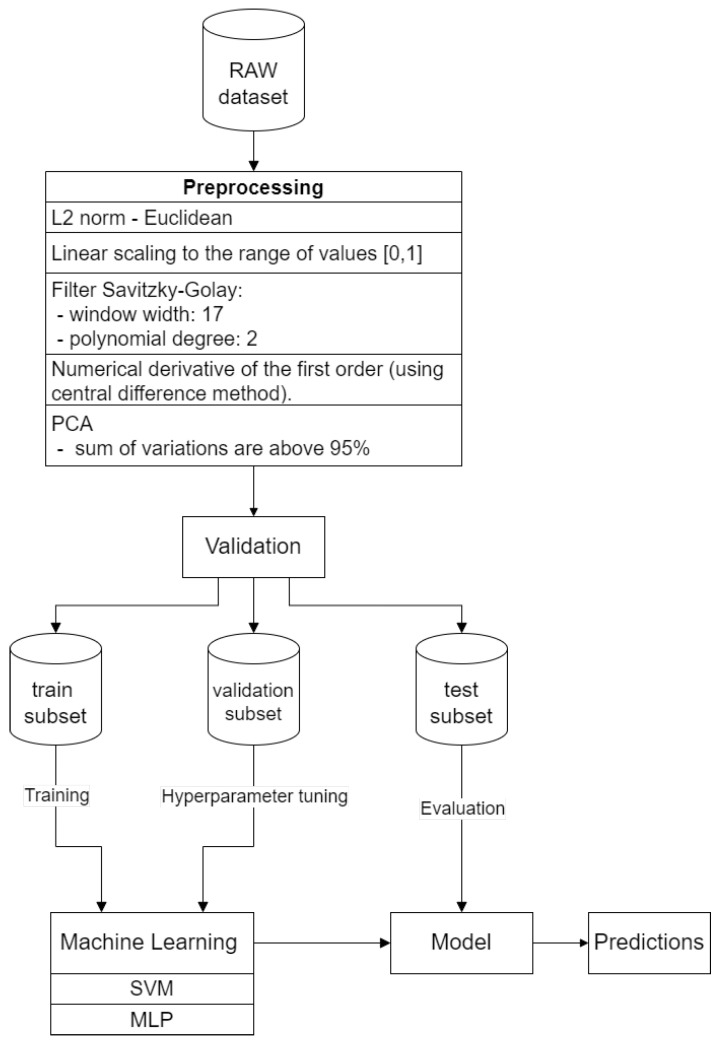
Prediction flowchart.

Figure 4 presents the flowchart that illustrates the applied method.

The first step was the normalization of spectral data for each pixel. This process led to the improvement of linear relationships between wavelengths and to noise reduction.

**Figure 5:**
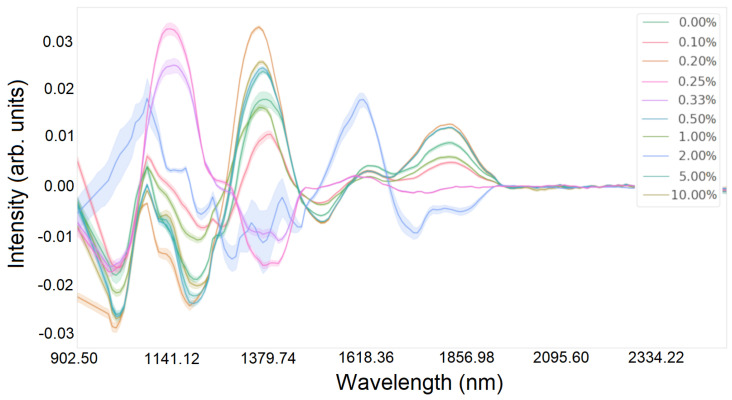
Spectral characteristics for all glucose concentrations after normalization and numerical differentiation.

**Figure 6:**
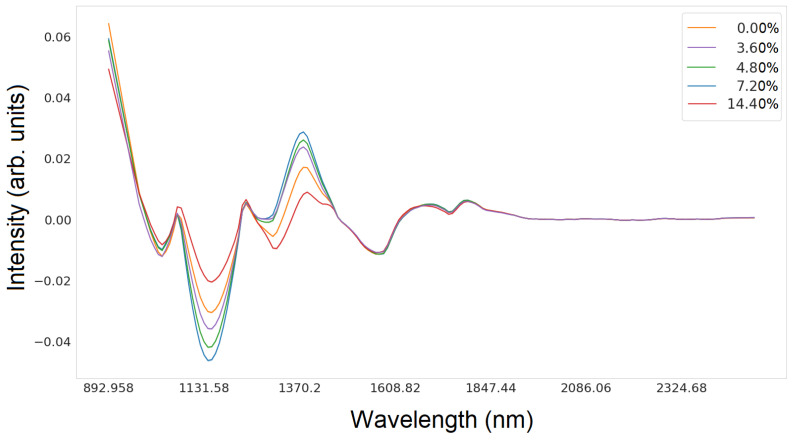
Spectral characteristics for all silicon concentrations after normalization and numerical differentiation.

A first-order numerical differentiation was applied using the central difference method for the data. Numerical differentiation of hyperspectral functions is particularly valuable when an analysis of the rates of change or the gradient of data variability is needed, which can facilitate new insights into fundamental trends or anomalies. However, due to the susceptibility to signal changes, it can amplify noise, leading to signal distortion. Therefore, it is advisable to apply a Savitzky–Golay filter in the preceding step to mitigate this effect [37]. Figure 5 and Figure 6 show the intensity distribution results of each wavelength after normalization and filtering. Figure 5 shows the results for glucose, and Figure 6 shows the results for silicon.

Based on hyperspectral data, principal components were determined to reduce dimensionality and, consequently, noise and distortions. Principal components are a linear combination of the original features, which makes their direct interpretation challenging.

Eigenvalues provide information about what part of the total variability is explained by a given principal component. The first principal component explains the largest part of the variance, the second principal component explains the largest part of the variance not explained by the previous component, and so on. As a result, each successive principal component explains a smaller part of the variance, meaning that successive eigenvalues are progressively smaller.

The total variance is the sum of the eigenvalues, which allows for calculation of the percentage of variability defined by each component. Consequently, for each successive component, the cumulative variability and the cumulative percentage of the variability can be computed.

**Figure 7:**
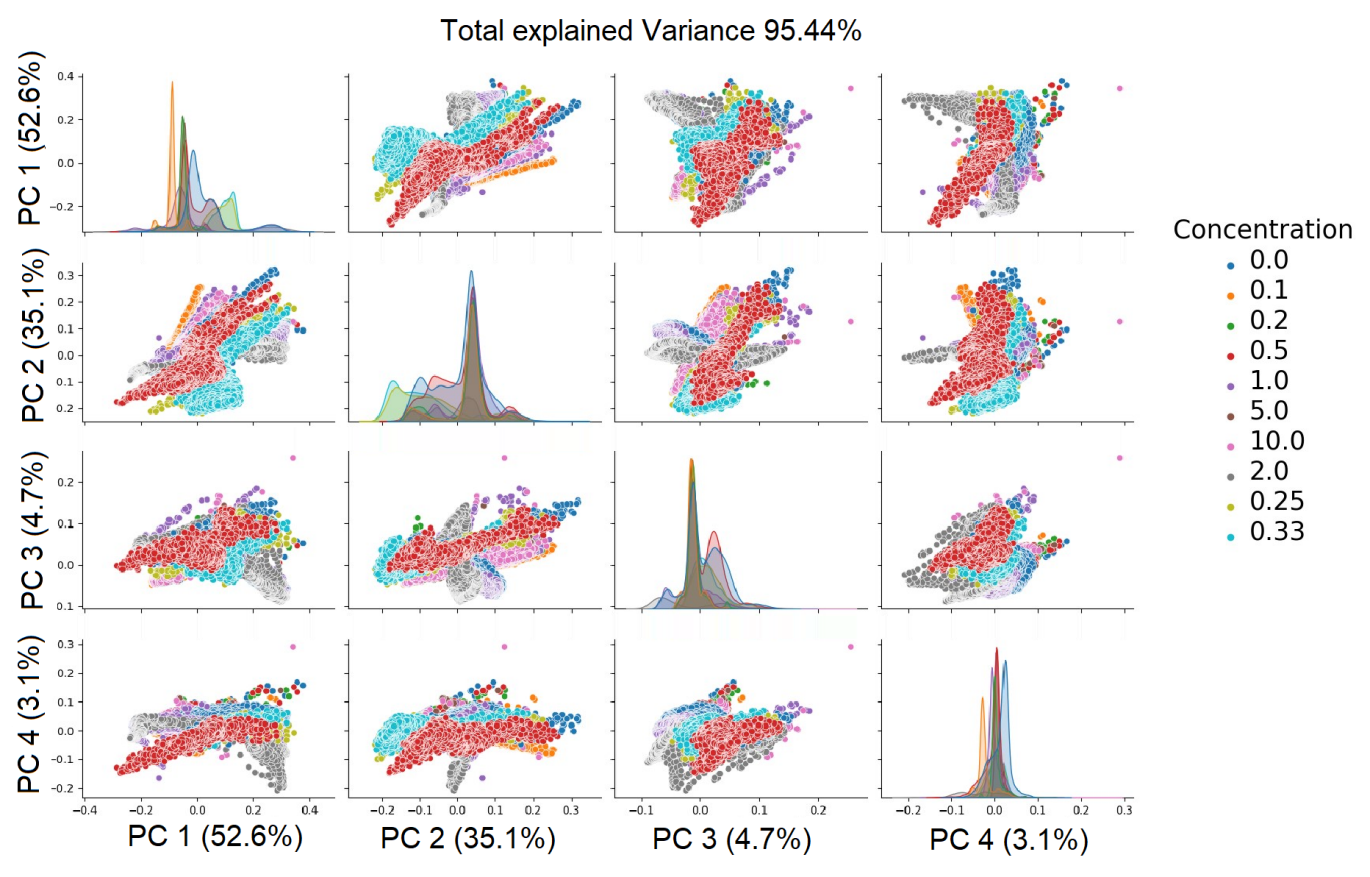
Scatter plot of principal components (PCs) of glucose.

**Figure 8:**
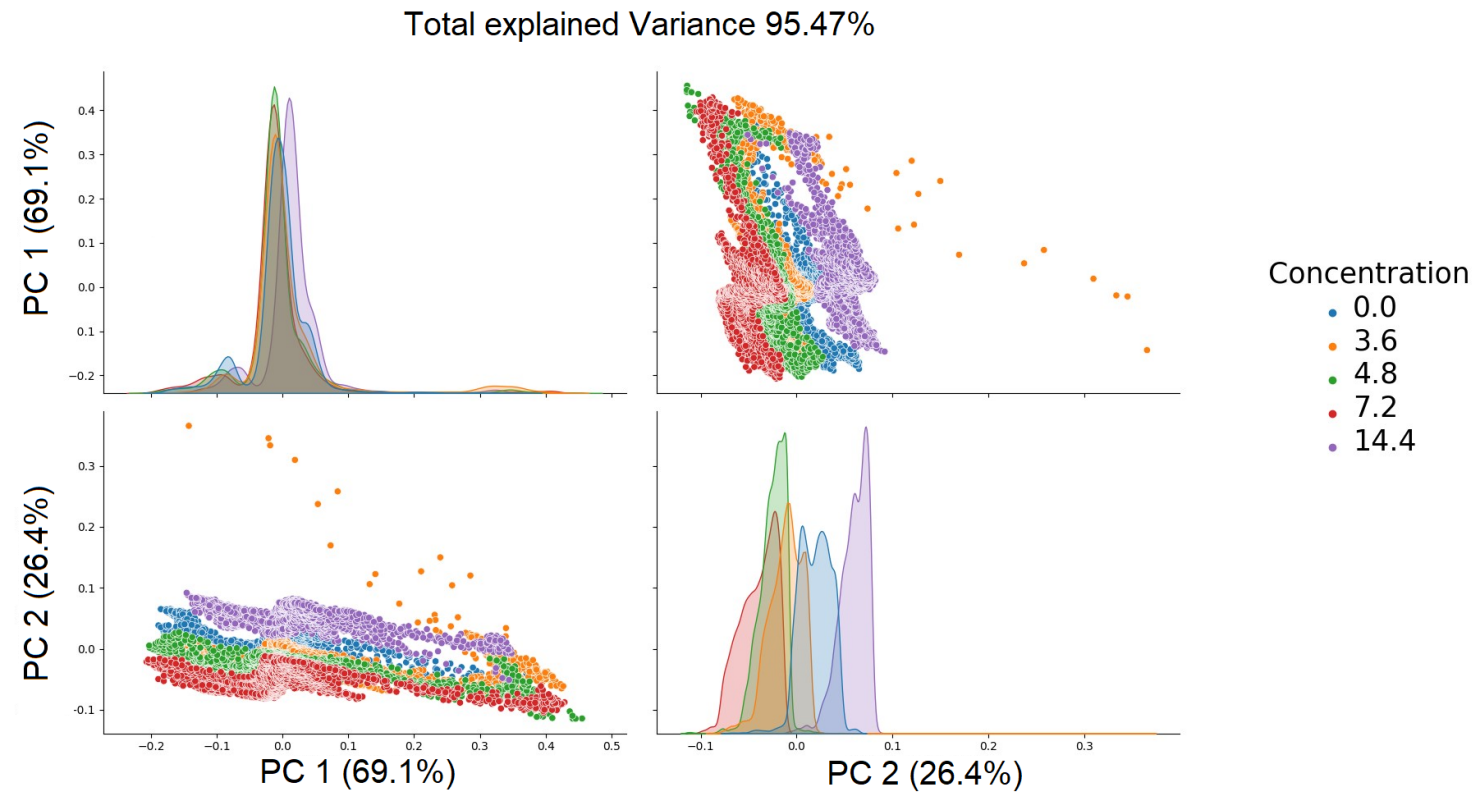
Scatter plot of principal components (PCs) of silicon.

Principal component analysis helps us to understand which original attributes enable accurate model classification. Visualization techniques, such as a biplot, can aid with understanding the relationships between variables and components. The relational matrix between the first five principal components is presented in Figure 7 and Figure 8.

The color indicates the concentration of each component. Clustering of the data depending on the concentration of the components can be clearly seen.

To achieve the highest ratio of conveyed information to the number of attributes, the decision was made to select the first three attributes. The choice was made to select the first four components due to the introduction of the greatest amount of conveyed information—the eigenvalues of these elements, in relation to the entire set, sum up to a total of 95%. The application of this method allowed for a reduction in the number of dimensions from 170 to 4 attributes for glucose and to 2 attributes for silicon. The results have been presented in Table 4 for a comparative analysis of the PCA outcomes between glucose and silicon.

#### 2.3.3. K-Fold Cross-Validation

K-fold cross-validation is a statistical method used to approximately assess the predictive ability of a created model. The process involves dividing the dataset into k equal subsets, where one of them is used for validation, while the remaining k-1 subsets are used to train and test the model. This method allows simultaneous training, validation, and testing of the model on different subsets of data.

K-fold cross-validation is particularly useful for mitigating the problem of overfitting during the learning process. It helps evaluate the stability of the model and its ability to generalize to different datasets.

#### 2.3.4. Prediction Methods

After preprocessing the collected data, two algorithms were used: the support vector machine regression (SVR) with a radial basis function (RBF) kernel and multilayer perceptron (MLP) with a ReLU activation function for the input and hidden layers.

Support vector regression (SVR) is a machine learning technique used in regression tasks. The primary goal of SVR is to find a regression function that has minimal deviation from the actual data while keeping the regression errors below a certain permissible level. Unlike traditional regression methods, SVR allows for adaptation to nonlinear relationships between variables.

MLP (multi-layer perceptron), a type of artificial neural network, can be used for both classification and regression tasks. MLP can model more complex, nonlinear relationships between inputs and outputs. MLP includes at least one hidden layer, allowing for the processing and detection of features at different levels of abstraction. This enables more advanced and intricate data representations, which can be beneficial for addressing more challenging problems.

The prepared models underwent k-fold cross-validation. K-fold cross-validation aims to assess the model’s quality during training to eliminate potential issues. The process involves dividing the training set into k subsets with an equal number of elements, where k-1 subsets are used for training/adapting the model, and one subset is used for validating the model.

## 3. Results

To determine which model has better predictions, we can analyze the mean absolute error (MAE), root-mean-square error (RMSE), or another appropriate evaluation metric. The lower the MAE or RMSE, the better the model’s predictions. Additionally, considering the units are in percentage (%), it us important to take into account the relative error.

Looking at Table 5 provided, we can analyze a summary of the metrics for model predictions for all models.

The hyperparameters for predicting glucose and silicon concentrations with optimal performance are defined in Table 6. Additionally, Table 7 presents the results for glucose concentration prediction across the entire sample range.

Notably, the MLP model demonstrates superior performance in this narrowed range. Conversely, for silicon prediction, the SVR model outperforms, as evidenced by the results in Table 8.

To evaluate the proposed solutions, we also conducted experiments using a classic method: linear regression. When evaluating silicon concentration, the evaluation error was about 5%. In the case of glucose, the error exceeded 10%. The results indicate a clear advantage for MLP (multi-layer perceptron) and SVR (support vector regression). The performance metrics suggest that both MLP and SVR outperform linear regression for accurately assessing the concentrations of silicon and glucose. This underscores the superiority of these models in comparison to the classic linear regression method, emphasizing their potential for more precise and reliable evaluations in this context.

## 4. Conclusions and Further Research

Through rigorous experimental validation and data analysis, the usefulness of hyperspectral cameras for detecting glucose and silicon particles has been demonstrated. The results emphasize the potential for real-time monitoring and analysis, contributing to better diabetes management, advanced material characterization, and medical diagnostics.

### Possibilities for Further Research and Development

Recognition of glucose and silicon concentrations can be expanded to include additional particles simultaneously within one model. The ability to consider a larger number of additional particles in various applications opens up new possibilities for future research. Additionally, extending efforts to create a model capable of simultaneously recognizing both the concentrations and types of particles present in varying intensities in the studied objects represents a promising path for broadening the implications of the method in various human activities—both in healthcare and materials science, among other areas, such as the analysis of artwork undergoing restoration.

In terms of future research and development regarding the ability to predict concentrations of different particles and chemical compounds using hyperspectral imaging technology, we provide the following observations.

In the context of healthcare, the ability to predict glucose and silicon concentrations using hyperspectral imaging has enormous potential to revolutionize non-invasive patient diagnostics. It can provide real-time information on glucose levels, offering a proactive approach to managing conditions such as diabetes. This technology could enable individuals to make informed lifestyle choices and allow healthcare professionals to conduct appropriate treatment processes on patients.

Beyond healthcare, the combination of hyperspectral imaging and predictive analysis could bring about a revolution in art restoration. The detailed information captured by hyperspectral imaging allows for a nuanced understanding of the materials used in artwork. Predicting silicon concentrations in pigments and substrates can assist art conservators with selecting the most appropriate and non-invasive restoration methods in order to preserve cultural heritage with unprecedented accuracy.

The future of this research lies not only in improving predictive models but also in developing portable hyperspectral imaging devices useful for artwork restoration when transportation is impossible due to legislative issues or the physical impossibility of moving the object.

The method may also find solutions in industry. The ability to predict residual sugar content is a significant step towards precision and consistency in production. Residual sugar, the unfermented sugars remaining in alcoholic beverages, plays a crucial role in determining the final flavor profile, perceived sweetness of the product, and the final alcohol content. The use of predictive technologies, especially in real-time analysis of brewing vats, can contribute to the precision and consistency of production. Our study intentionally omitted investigations into very low levels of silicon. Our research’s primary focus was on evaluating the efficacy and performance of the support vector regression (SVR) and multi-layer perceptron (MLP) algorithms. We aimed to assess how well these algorithms could handle moderate to high levels of silicon by concentrating on a specific range of silicon concentrations. We plan to extend our experimental scope to include studies with silicon concentrations more closely aligned with medical applications.

## Figures and Tables

**Table 1:** Summary review of glucose detection methods.

Ref	Objectives and Proposed Method	Advantages and Disadvantages
[11]	**NIR spectroscopy**: A method using visible near-infrared optical biosensor for non-invasive blood glucose monitoring.	(Adv) The biosensor offers a noninvasive and continuous blood glucose monitoring solution that provides a more comfortable and user-friendly alternative to traditional methods. (Dis) The use of a short data acquisition time window (10 s) may raise questions about the biosensor’s ability to capture dynamic glucose changes accurately, especially in situations with rapid fluctuations.
[12]	**NIR spectroscopy**: A noninvasive measurement method is proposed and examined to continuously predict blood glucose content using near-infrared diffuse reflection difference spectra.	(Adv) The method provides a clear understanding of the blood glucose prediction process, aiding in the refinement and optimization of the prediction model. (Adv) Identification of the main factors contributing to prediction errors allows for targeted improvements, enhancing the overall accuracy and reliability of the prediction method. (Dis) The presence of unknown disturbances affecting prediction accuracy indicates potential limitations to the model’s robustness. (Dis) The introduction of imaginary components and the separation of glucose contributions may add complexity to the prediction model.
[13]	**MIR spectroscopy**: A method using mid-infrared absorption spectroscopy for performing non-invasive blood glucose measurements is presented.	(Adv) The method relies on only a few selected wave numbers (1050 cm^−1^, 1070 cm^−1^, and 1100 cm^−1^). (Adv) The technique allows for unconditional blood glucose level measurements in vivo, eliminating the need for calibration. (Dis) There is no mention of experimental validation or actual measurement results.
[14]	**FIR spectroscopy**: A method using FIR spectroscopy for performing noninvasive blood glucose measurements is presented.	(Adv) The proposed strategy aims to enhance the selectivity of THz spectroscopy for aqueous biological sample detection, overcoming a known limitation to traditional THz spectroscopy. (Adv) In the proposed method, calibration of the device is not required. (Dis) The proposed estimation of the glucose level is difficult because of the strong absorption of water and due to the low level of penetration.
[15]	**Raman spectroscopy**: The main objective is to identify glucose fingerprint peaks in in vivo transcutaneous Raman measurements.	(Adv) The identification of glucose fingerprint peaks enhances the selectivity of Raman measurements for glucose, providing a more accurate and specific method for monitoring glucose levels. (Adv) The noncontact off-axis system minimizes probe instability and reduces the impact of specular Rayleigh reflection, improving the overall reliability of the Raman measurements. (Adv) The use of intraspectrum band area ratios allows for normalized changes to glucose Raman bands, reducing the influence of measurement artifacts and ensuring the accuracy of glucose concentration measurements. (Dis) The text mentions challenges such as lowering the maximum glucose concentration, reducing integration time, and miniaturizing the system for practical use. These challenges highlight the need for further technological development.
[16]	**Raman spectroscopy**: The primary objective is to develop a table-top confocal, critical-depth Raman spectrometer designed for home use by patients with diabetes.	(Adv) The system demonstrates an extended calibration stability of at least 10 days, providing a reliable and consistent analytical tool for users over an extended period. (Adv) The critical-depth Raman system exhibits performance metrics, such as MARD and consensus error grid readings, that are comparable to current invasive continuous monitoring devices, suggesting its potential as a non-invasive alternative. (Dis) The current calibration is based on individual partial least squares (PLS) models for each patient, and further development is required to establish a global calibration strategy accommodating individual patient characteristics and optical properties of the skin. (Dis) The application of machine learning techniques, categorization, and nonlinear regression models for calibration requires further investigation, and challenges to optimizing these approaches may be encountered during the development of the critical-depth Raman system.

**Table 2:** Collective statistical description of spectral data for all glucose concentrations.

Wavelength [nm]	σ	Skewness	Kurtosis	Correlation
892.958	19,888.18	2.349	3.536	0.125
902.503	675.41	−0.156	−0.756	−0.328
912.048	901.58	0.362	−1.721	−0.335
921.593	1109.65	0.408	−1.629	−0.333
931.138	1273.82	0.431	−1.573	−0.329
…	…	…	…	…
2467.85	682.89	0.296	−1.634	−0.337
2477.39	689.39	0.296	−1.687	−0.338
2486.94	686.49	0.292	−1.723	−0.338
2496.48	686.56	0.277	−1.758	−0.339
2506.03	690.22	0.255	−1.761	−0.337

**Table 3:** Collective statistical description of spectral data for all silicon concentrations.

Wavelength [nm]	σ	Skewness	Kurtosis	Correlation
892.958	39.08	1.169	2.381	−0.5
902.503	49.19	0.862	1.519	−0.579
912.048	177.44	1.327	12.857	−0.237
921.593	293.12	1.197	12.355	−0.116
931.138	360.53	1.256	13.576	−0.105
…	…	…	…	…
2467.85	39.25	0.516	−0.055	−0.715
2477.39	39.16	0.538	0.1797	−0.713
2486.94	39.99	0.438	−0.110	−0.699
2496.48	38.46	0.488	−0.111	−0.708
2506.03	38.51	0.517	−0.025	−0.710

**Table 4:** Principal component analysis results.

	Glucose	Silicon
Principal Components	4	2
Explained Variance	95.44%	95.47%

**Table 5:** Quality metrics for different models for predicting silicon and glucose concentrations.

	Model	RMSE	R^2^	MAE
Silicon	SVR	0.0070	0.99	0.079
	MLP	0.0085	0.99	0.045
Glucose	SVR	0.0036	0.99	0.052
	MLP	0.0020	0.99	0.014

**Table 6:** Hyperparameters of MLP for glucose.

Hyperparameter	Value for Glucose	Value for Silicon
Hidden layers	2	2
Hidden size	248	51
Learning rate	0.003	0.01
Epochs	4	6
Batch size	113	6

**Table 7:** Glucose concentrations by MLP and SVR.

True Value	MLP Prediction	SVR Prediction
0.00%	0.000 ± 0.0006	0.073 ± 0.0731
0.10%	0.100 ± 0.0005	0.129 ± 0.0289
0.20%	0.200 ± 0.0005	0.185 ± 0.0156
0.25%	0.250 ± 0.0009	0.222 ± 0.0277
0.33%	0.330 ± 0.0008	0.281 ± 0.0492
0.50%	0.500 ± 0.0008	0.409 ± 0.0912
1.00%	1.000 ± 0.0009	0.906 ± 0.0938
2.00%	2.000 ± 0.0016	2.095 ± 0.0946
5.00%	5.000 ± 0.0010	4.901 ± 0.0988
10.00%	10.000 ± 0.0009	9.915 ± 0.0846

**Table 8:** Silicon concentrations by MLP and SVR.

True Concentration	MLP Prediction	SVR Prediction
0.00%	0.002 ± 0.0081	0.097 ± 0.0012
3.60%	3.607 ± 0.0171	3.500 ± 0.0017
4.80%	4.806 ± 0.0207	4.809 ± 0.0014
7.20%	7.209 ± 0.0308	7.300 ± 0.0002
14.40%	14.420 ± 0.0621	14.306 ± 0.0015

## Data Availability

Data is contained within the article.

## List of Acronyms

Glc	Glucose
HSI	Hyperspectral Imaging
MAE	Mean Absolute Error
MIR	Mid-Infrared
MLP	Multi-Layer Perceptron
NIR	Near-Infrared
PC	Principal Component
PCANN	PCA Neural Network
RMSE	Root-Mean-Square Error
Si	Silicon
SVM	Support Vector Machine
SVR	Support Vector Regression
UV	Ultraviolet
VIS	Visible Light
